# USP33, a new player in lung cancer, mediates Slit-Robo signaling

**DOI:** 10.1007/s13238-014-0070-z

**Published:** 2014-07-02

**Authors:** Pushuai Wen, Ruirui Kong, Jianghong Liu, Li Zhu, Xiaoping Chen, Xiaofei Li, Yongzhan Nie, Kaichun Wu, Jane Y. Wu

**Affiliations:** 1 State Key Laboratory of Brain and Cognitive Science, Institute of Biophysics, Chinese Academy of Sciences, Beijing, 100101 China; 2 Department of Neurology, Center for Genetic Medicine, Lurie Cancer Center, Northwestern University Feinberg School of Medicine, 303 E. Chicago Ave., Chicago, IL 60611 USA; 3 Department of Thoracic Surgery, Tangdu Hospital, Fourth Military Medical University, Xi’an, 710038 China; 4 State Key Laboratory of Cancer Biology and Xijing Hospital of Digestive Diseases, Fourth Military Medical University, Xi’an, 710032 China; 5 University of Chinese Academy of Sciences, Beijing, 100049 China; 6 Department of Pathophysiology, Liaoning Medical University, Jinzhou, 121001 China

**Keywords:** USP33, Slit, Robo, lung cancer, Slit-Robo signaling, tumor suppressor, prognostic marker

## Abstract

Ubiquitin specific protease 33 (USP33) is a multifunctional protein regulating diverse cellular processes. The expression and role of USP33 in lung cancer remain unexplored. In this study, we show that USP33 is down-regulated in multiple cohorts of lung cancer patients and that low expression of USP33 is associated with poor prognosis. USP33 mediates Slit-Robo signaling in lung cancer cell migration. Downregulation of USP33 reduces the protein stability of Robo1 in lung cancer cells, providing a previously unknown mechanism for USP33 function in mediating Slit activity in lung cancer cells. Taken together, USP33 is a new player in lung cancer that regulates Slit-Robo signaling. Our data suggest that USP33 may be a candidate tumor suppressor for lung cancer with potential as a prognostic marker.

**Electronic supplementary material:**

The online version of this article (doi:10.1007/s13238-014-0070-z) contains supplementary material, which is available to authorized users.

## Introduction

Lung cancer is the most frequently diagnosed of all cancers and responsible for approximately 1.38 million deaths worldwide every year with an overall 5-year survival rate ~15% (Ferlay et al., 2010). Although surgery, radiation, chemotherapy, and targeted treatments have been developed, these treatments, even in combinations, are far from satisfactory. A number of tumor suppressor genes have been discovered in lung cancer, including TP53, p16, LKB1/STK11, NF1, RASSF1, APC, BRG1, PTEN, and RB (reviewed in Cooper et al., 2013; Herbst et al., 2008). A variety of vulnerability genes have been identified recently (Kim et al., 2013). However, endogenous mechanisms that suppress lung cancer invasion and metastasis remain largely unknown.

Several neuronal guidance molecules have been implicated in cancer invasion and metastasis (Ballard and Hinck, 2012). Originally identified in *Drosophila* (Rothberg et al., 1988), Slit genes encode a family of secreted proteins that act as neuronal guidance molecules (Bashaw et al., 2000; Brose et al., 1999; Li et al., 1999; Simpson et al., 2000; Wu et al., 1999). Recent studies suggest the involvement of Slit genes in invasion and metastasis of different types of cancers (e.g., Biankin et al., 2012; Brantley-Sieders et al., 2011; Dallol et al., 2003; Yuasa-Kawada et al., 2009a; Tie et al., 2010). Slit acts by binding to a single-pass transmembrane protein Roundabout (Robo). Our previous data have shown that one of the Robo-interacting proteins that mediate Slit-Robo signaling is ubiquitin specific protease 33 (USP33), also known as von Hippel-Lindau (VHL)-interacting deubiqutinating enzymes 1, (VDU1) (Yuasa-Kawada et al., 2009a, b). As a member of ubiquitin-specific protease family, USP33 was initially discovered as a substrate protein binding to VHL E3 ligase (Li et al., 2002). USP33 is required for Slit signaling in midline commissural axon guidance (Yuasa-Kawada et al., 2009b). In addition, USP33 is involved in Slit signaling in inhibiting breast caner cell migration (Yuasa-Kawada et al., 2009a), suggesting that USP33 may play an important role in cancer invasion and metastasis. However, the role and mechanism of USP33 in Slit signaling in lung cancer remain unknown.

In this study, we demonstrate that USP33 is required for Slit inhibition of lung cancer cell migration. USP33 regulates the level of Robo receptor in lung cancer cells. This is distinct from the USP33 activity in neurons and breast cancer cells (Yuasa-Kawada et al., 2009a, b). In addition, USP33 appears to have a tumor suppressor function in lung cancer because low expression of USP33 correlates strongly with poor survival of lung cancer patients.

## Results

### USP33 expression is down-regulated in lung cancer

**Figure 1:**
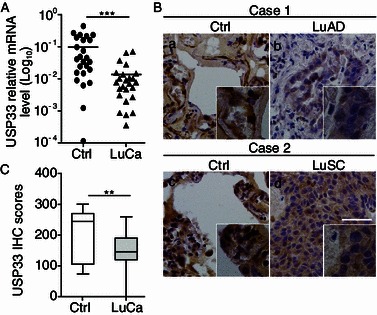
**USP33 expression is down-regulated in human lung cancer samples**. (A) USP33 mRNA level in 25 pairs of lung cancer and adjacent non-cancer lung control tissues. Values shown (Y axis represents relative expression units) are normalized to GAPDH. ***P < 0.001. (B) Immunohistochemical staining of USP33 in lung cancer and control tissues. a and c, strong positive immunoreaction for USP33 is detected in the cytoplasm. b, USP33-positive case of lung adenocarcinoma (LuAD). d, USP33-positive case of lung squamous cell carcinoma (LuSC). Bar: 50 μm. (C) Quantification of USP33 protein expression in lung cancer (*n* = 25) and the adjacent control non-cancer lung tissues (Ctrl) (*n* = 25). The USP33-immunohistochemistry scores are calculated as follows: USP33 immunohistochemistry score = (% of positive tumour cells) × the staining intensity. USP33 protein expression in lung cancer samples is significantly lower than in the control lung tissues (**, *P* < 0.01)

To identify new players and evaluate the potential role of USP33 in lung cancer, we measured levels of USP33 expression in a panel of lung cancer samples with the matched adjacent non-tumor lung tissue controls. The level of USP33 mRNA was analyzed by quantitative RT-PCR. USP33 expression was significantly lower in lung cancer samples, as compared with adjacent non-tumor lung tissues (*P* < 0.001) (Fig. 1A). We next examined USP33 protein expression in lung cancer samples by immunohistochemical staining using an antibody specific for USP33 (Fig. 1B; see Fig. S1 for the specificity of anti-USP33). The USP33 staining signals in the normal lung tissues showed a range from 73.33 to 300.00 (with the mean value as 198.18), whereas those in the lung cancer samples varied from 0.00 to 266.00 (with the mean value as 142.38). The USP33 expression levels in lung cancer samples were significantly lower than those in paired adjacent non-tumor lung tissues (*P* < 0.01; Fig. 1B and 1C).

**Figure 2:**
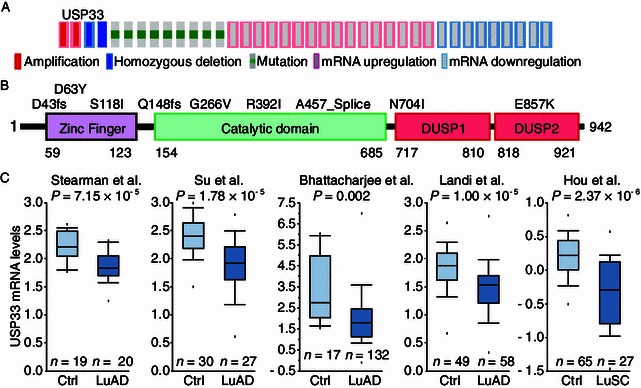
**USP33 expression is reduced in human lung cancer samples obtained from public databases**. (A and B) Genetic alterations and mRNA expression changes of the USP33 in the TCGA dataset of lung cancer samples. USP33 genes are represented as rows, and individual cases or patients are represented as columns. Genetic alterations are color-coded with red indicating amplification; light blue, homozygous deletion; green box, somatic mutation; pink frame, mRNA upregulation; and blue frame, mRNA downregulation. These oncoprints are based on data obtained from the Stand Up to Cancer cBio portal (http://cbio.mskcc.org/su2c-portal/). Higher magnification images are shown in the insets (B). (C) Five lung cancer gene expression studies are analyzed using Oncomine (http://www.oncomine.org). USP33 is significantly down-regulated at the mRNA level in lung cancer samples as compared to the control lung tissues in all 5 datasets (Stearman et al., Su et al., Bhattacharjee et al., Landi et al., and Hou et al.). Box and whisker plots: dots represent maximum and minimum values, whiskers show 90th and 10th percentiles, boxes show 75th and 25th percentiles, and the line indicates the median value. *P*-values were computed by Oncomine software using Student’s *t*-test. Ctrl: control lung tissue samples; LuAD: lung adenocarcinoma; LuSC: lung squamous cell carcinoma

To investigate what fraction of lung cancer patients may have USP33 expression changes, we analyzed a larger dataset obtained from cBioPortal for Cancer Genomics (http://cbioportal.org) (Gao et al., 2013). A total of 554 cases were examined in this cohort. USP33 mRNA down-regulation was detected in 9 lung cancer cases with homozygous deletion in 2 additional cases and somatic mutations in another 9 cases (Fig. 2A and 2B). Notably, four USP33 mutations reside inside the catalytic domain of USP33 (Fig. 2B). Thus, approximately 4% of these lung cancer cases showed homozygous deletion, somatic mutations or decreased mRNA expression. In addition, somatic USP33 missense mutations were detected in of other cohorts of lung adenocarcinoma (2.7%–6.2%) and lung squamous cell carcinoma (3.4%) samples (Fig. S2). USP33 expression profiles were further examined in datasets from Oncomine database (http://www.oncomine.org/) (Rhodes et al., 2004), which contained mRNA profiling data from 5 independent cohorts of lung cancer samples. Remarkably, USP33 mRNA levels were significantly lower in lung cancer samples as compared with the control samples in all 5 datasets (Fig. 2C). Together, these results show a highly consistent pattern of down-regulation of USP33 expression in lung cancer samples.

### USP33 expression levels correlate with clinical outcomes

**Figure 3:**
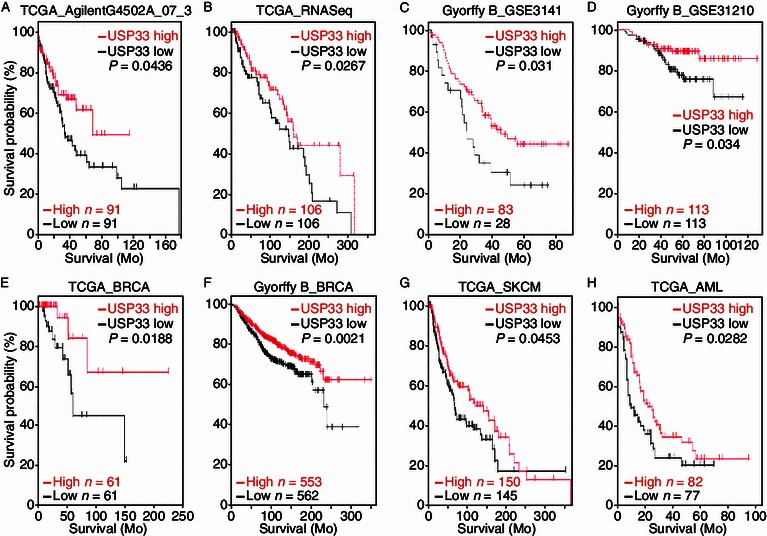
**Low USP33 expression correlates with poor patient survival in multiple cancer datasets**. Kaplan-Meier curves showing the overall survival analysis in patients with high and low expression of USP33 from eight public tumor datasets: (A) TCGA lung data set (AgilentG4502A_07_3 array, *n* = 91 for USP33 low, *n* = 91 for USP33 high; *P* = 0.0436 with log-rank analysis); (B) TCGA lung data set (IlluminaHiSeq_RNASeqV2 array, *n* = 106 for USP33 low, *n* = 106 for USP33 high; *P* = 0.0267 with log-rank analysis); (C) Lung cancer dataset (GSE3141) was analyzed by KM plotter. The data were dichotomized at the lower quantile value into high and low expressing groups (*n* = 28 for USP33 low, *n* = 83 for USP33 high; *P* = 0.031 with log-rank analysis); (D) Lung cancer dataset (GSE31210) was analyzed by KM plotter. The data were dichotomized at the median value into high and low expressing groups (*n* = 113 for USP33 low, *n* = 113 for USP33 high; *P* = 0.034 with log-rank analysis). (E) TCGA breast carcinoma data set (*n* = 61 for USP33 low, *n* = 61 for USP33 high; *P* = 0.0188 with log-rank analysis); (F) Breast cancer data were analyzed by KM plotter. The data were dichotomized at the median value into high and low expressing groups (*n* = 562 for USP33 low, *n* = 553 for USP33 high; *P* = 0.0021 with log-rank analysis); (G) TCGA skin cutaneous melanoma data set (*n* = 145 for USP33 low, *n* = 150 for USP33 high; *P* = 0.0453 with log-rank analysis); (H) TCGA acute myeloid leukemia data set (*n* = 77 for USP33 low, *n* = 82 for USP33 high; *P* = 0.0282 with log-rank analysis)

To examine the relationship between USP33 expression and the clinical outcome, we analyzed association between USP33 expression and patient survival by examining publically available microarray profiling datasets for lung cancer. We generated Kaplan-Meier (KM) survival curves from The Cancer Genome Atlas (TCGA) datasets (Sanborn et al., 2011; Zhu et al., 2009) and online KM-Plotter database (Gyorffy et al., 2010; Gyorffy et al., 2013). For each dataset, lung cancer patients were classified into two groups based on the expression level of the USP33 gene. Kaplan-Meier analysis was used to evaluate survival differences between the group with high USP33 expression and that with low USP33 expression. In all four lung cancer datasets in which survival data are available, higher USP33 expression correlates with longer overall survival of patients (Fig. 3A–D).

We further interrogated the association of USP33 expression with patient survival in other types of cancers. Similarly, lower expression of USP33 correlated with poorer survival in a range of other types of cancers, including breast cancer (BRCA, Fig. 3E and 3F), melanoma (SKCM, Fig. 3G) and acute myeloid leukemia (AML, Fig. 3H). These data suggest that USP33 may act as a tumor suppressor gene in a variety of human cancers.

### USP33 is required for Slit signaling in inhibiting lung cancer cell migration

Previous studies report that Slit expression is reduced in different types of cancer, including lung cancer and breast cancer (Dallol et al., 2002) and that Slit plays a role in suppressing lung cancer progression (Tseng et al., 2010). Our previous work indicates that USP33 is required for Slit signaling in breast cancer (Yuasa-Kawada et al., 2009a). To understand the mechanism of USP33 function in lung cancer cells, we investigated the role of USP33 in mediating Slit suppression of cancer cell migration.

**Figure 4:**
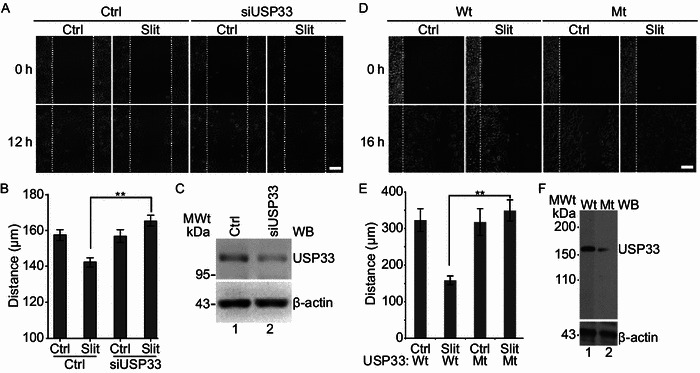
**USP33 regulates Slit activity in suppressing lung cancer cell migration dependent on its catalytic domain**. (A) Imaging of wound healing of H1299 cells transfected with Ctrl siRNA or siUSP33 and (D) wild-type or mutant (C163A) USP33 was performed in the presence of mock-control (Ctrl) or Slit. Wound scratches were made so that the cells at the wound edge were migrating toward the right side in each panel. Images taken at two time points after the wound scratching are shown. The white dotted lines mark the wound edge at 0 h (Scale bar: 100 μm). (B and E) Quantification of the distance of cell migration in the direction toward the center of the wound. Data are presented as the mean ± SEM. **, *P* < 0.01 by Mann-Whitney test. (C) Western blot analysis showing that siUSP33, but not Ctrl siRNA, suppressed expression of endogenous USP33, in H1299 cells. Beta-actin was used as an internal control. (F) The expression of wild-type and mutant (C163A) USP33 in H1299 cells were confirmed by Western blot. Beta-actin was used as an internal control

We first tested the involvement of USP33 in Slit inhibition of lung cancer cell migration in a wound healing assay. H1299 lung cancer cells were treated with either mock control or Slit containing media following wound formation on a monolayer culture of H1299 cells. Slit treatment led to a significant reduction in cell migration of H1299 lung cancer cells. Upon down-regulation of USP33 by a specific siRNA against USP33 (siUSP33), the effect of Slit in suppressing lung cancer cell migration was abolished (Fig. 4A–C), indicating that USP33 is required for Slit signaling in lung cancer cells. Neither Slit treatment nor USP33 downregulation affected cell proliferation or cell cycle in these experiments (Fig. S3 and S4).

To test if the deubiquitinating activity of USP33 plays a role in mediating Slit activity in lung cancer cells, we used a construct of USP33 mutant that contained a point mutation, C163A, in the catalytic domain of USP33 and was capable of acting in a dominant negative fashion (Yuasa-Kawada et al., 2009b). Following transfection of H1299 cells with either wild type (Wt) USP33 or the C163A-mutant USP33 (Mt), H1299 cells were treated with control- or Slit-containing medium (Fig. 4D–F). Expression of the C163A-mutant USP33 abolished Slit suppression of H1299 cell migration, supporting that deubiquitinating activity of USP33 is important in mediating Slit activity in inhibiting lung cancer cell migration.

### USP33 regulates the stability of Robo1

**Figure 5:**
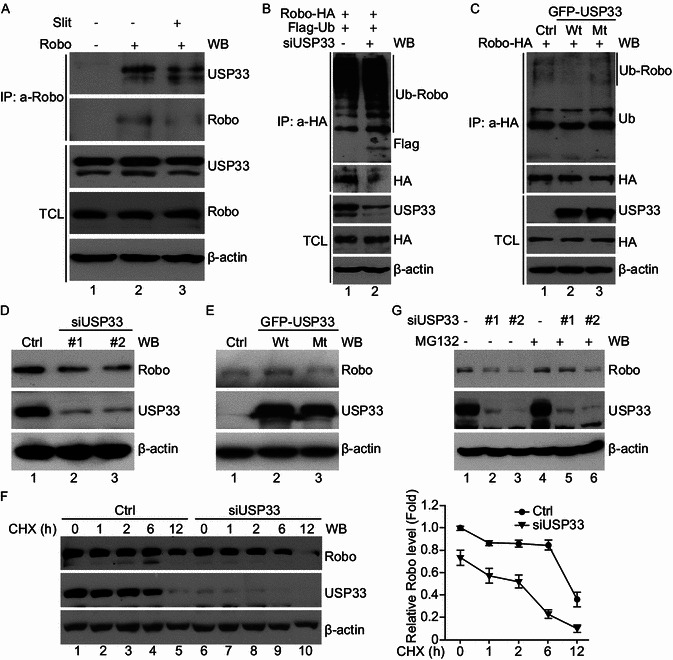
**USP33 interacts with Robo1 and affects the Robo1 stability in lung cancer cells**. (A) Interaction of the endogenous Robo1 and USP33 proteins in H1299 cells. Coimmunoprecipitation was carried out using either control IgG or anti-Robo1 antibodies in the presence of mock-control (Ctrl) or Slit. Immunoprecipitated proteins were analyzed by Western blotting with anti-USP33. (B) Deubiquitination assay was performed in H1299 cells transfected with Robo-HA, Flag-ubiquitin, Ctrl siRNA or siUSP33. Co-immunoprecipitation was carried out with anti-HA antibodies after MG132 (20 μmol/L) treatment for 6 h. Immunoprecipitated proteins were examined by Western blotting using corresponding antibodies as indicated. (C) Robo1 deubiquitination was examined in H1299 cells following transfection with Robo-HA, control, wild type or mutant USP33 plasmids. Coimmunoprecipitation was done with anti-HA antibodies after MG132 (20 μmol/L) treatment for 6 h. Immunoprecipitated proteins were used in Western blotting using corresponding antibodies as indicated. (D and E) Western blot analysis showing that the levels of the endogenous USP33 and Robo1 proteins in H1299 cells following transfection with siRNAs targeting USP33 #1, USP33#2, and Ctrl siRNA (D) or control, wild type or mutant USP33 plasmid (E). Beta-actin was used as the internal control. (F) H1299 cells were transfected with siUSP33 and Ctrl siRNA and treated with CHX (50 μg/mL) for variable lengths of time. The turnover of endogenous Robo1 was determined by Western blot. Beta-actin was used as the internal control. (G) H1299 cells transfected with siUSP33 and Ctrl siRNA were left untreated or treated with MG132 (20 μmol/L) for 6 h, then proteins were extracted and subjected to Western blotting analyses. Beta-actin was used as the internal control

**Figure 6:**
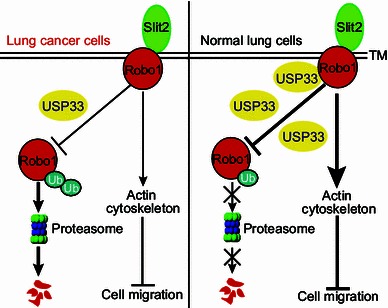
**A diagram illustrating our working model for USP33 function in Slit signaling in lung cancer cells**. In the normal lung cells, Slit2 binds to Robo1 and activates downstream molecules, modulating the actin cytoskeleton and inhibiting cell migration. Deubiquitinylating enzyme USP33 reduces the ubiquitination and degradation of Robo1, thereby stabilizing Robo1 protein. However, in lung cancer cells, reduced USP33 expression leads to increased ubiquitination and degradation of Robo1, inactivating Slit signaling in inhibiting lung cancer cell migration

In breast cancer cells, USP33 acts to redistribute Robo1 from the intracellular compartment to cell surface without affecting the total level of Robo1 protein (Yuasa-Kawada et al., 2009a). We asked if USP33 in lung cancer cells acted in a similar manner as in breast cancer cells. In lung cancer cells, USP33 interacted with Robo1, similar to the observation in the breast cancer cells, as detected by co-immunoprecipitation experiments (Fig. 5A). Knocking-down USP33 in H1299 cells increased the level of ubiquitinylated Robo1 in the presence of MG132, an inhibitor of the ubiquitin proteasome system (Fig. 5B). Overexpression of wild-type USP33, but not catalytically inactive C163A mutant of USP33, decreased the ubiquitination of Robo1 (Fig. 5C). Unexpectedly, down-regulation of USP33 by specific siRNAs in H1299 cells led to a decrease in the Robo1 protein level, in contrast to the findings in breast cancer cells (Fig. 5D; Yuasa-Kawada et al., 2009a). Conversely, overexpression of wild-type USP33, not the C163A mutant USP33, in H1299 cells increased the levels of Robo1 (Fig. 5E). To examine how USP33 regulates Robo1 protein stability, H1299 cells were transfected with control siRNA (Ctrl) or siUSP33, and treated with cycloheximide (CHX, an inhibitor of protein synthesis) for different periods of time before the preparation of cell lysates. The Robo1 protein was detected by Western blotting analyses of cell lysates. At 6 h after CHX treatment, the Robo1 level decreased; and by 12 h following CHX treatment, Robo1 protein was almost completely degraded in H1299 cells transfected with siUSP33 compared with Ctrl siRNA, indicating that knocking-down USP33 shortens the half-life of Robo1 (Fig. 5F). The decrease of Robo1 protein level induced by siUSP33 was blocked by treatment with proteasome inhibitor MG132 (Fig. 5G), suggesting that Robo1 was degraded mainly through the ubiquitin-proteasome pathway in H1299. These results indicate that in lung cancer cells, USP33 stabilizes Robo1 protein, preventing it from ubiquitin-proteasome mediated degradation (Fig. 6).

## Discussion

Our results provide evidence that USP33 is a new player in lung cancer that regulates Slit-Robo signaling. We demonstrate that expression of USP33 is down-regulated in lung cancer and associated with clinical outcomes. USP33 mediates the Slit activity in inhibiting lung cancer cell migration in a manner that is dependent on its catalytic domain. Furthermore, USP33 stabilizes Robo1 protein by inhibiting proteasome-dependent degradation. These results have revealed a previously unknown role of USP33 in mediating Slit-Robo signaling in lung cancer cells.

USP33 has been implicated in a number of cellular processes (Berthouze et al., 2009; Buus et al., 2009; Curcio-Morelli et al., 2003; Li et al., 2013; Shenoy et al., 2009). Our study provides, for the first time, evidence that USP33 is a lung cancer associated gene and that its expression is reduced in lung cancer tissue samples. This down-regulated expression of USP33 was observed in multiple microarray datasets of lung cancer and confirmed by qRT-PCR and immunohistochemical analysis. The higher expression of USP33 is associated with better prognosis of the lung cancer patients. The human USP33 gene is located at the chromosome 1p31.1, the region that shows approximately 50% allelic loss in the NSCLC. In this region, a few tumor suppressor genes have been identified (Girard et al., 2000). For example, DnaJ-like heat shock protein (HLJ1) as a tumor suppressor locating at this region has been identified to inhibit lung cancer cell proliferation, anchorage-independent growth, tumorigenesis, cell motility, and invasion (Tsai et al., 2006). The protein kinase cAMP-dependent catalytic beta (PRKACB) is downregulated in NSCLC tissues, and upregulation of PRKACB prevents the progression of NSCLC (Chen et al., 2013). In the present study, our results suggest that USP33 is a critical player in lung cancer and can be a potential prognostic biomarker for lung cancer progression.

Recently, altered expression of different genes in the USP family has been associated with different types of cancers including lung cancer. For example, USP44 was downregulated in human lung adenocarcinoma (Zhang et al., 2012). Patients with lower levels of the USP44 enzyme showed a significantly reduced overall survival (Zhang et al., 2012). The intragenic homozygous rearrangements and deletions of the nuclear deubiquitinase (BAP1) have been found in lung carcinoma cell lines (Ventii et al., 2008). USP1/UAF1 may act as an oncogene, because the inhibitors of USP1/UAF1 act synergistically with cisplatin in inhibiting cisplatin-resistant cell proliferation in NSCLC (Chen et al., 2011). Data presented in this study support that USP33 may be a new tumor suppressor gene in lung cancer.

In human lung cancer, low expression of Slit2 is associated with late-stage disease and poor patient survival (Tseng et al., 2010). Robo1-deficient mice exhibit bronchial epithelial hyperplasia and focal dysplasia, pathological features associated with early-stage lung cancer (Xian et al., 2001). In our study, Slit2 significantly inhibits lung cancer cell migration, supporting that Slit-Robo signaling suppresses lung cancer.

Our previous study has demonstrated that Slit inhibits breast cancer cell migration in a mechanism dependent on USP33 (Yuasa-Kawada et al., 2009a). Our current work shows that in lung cancer cells, USP33 also interacts with Robo1 and is required for Slit signaling in inhibiting lung cancer cell migration. Ubiquitin-mediated modification plays a critical role in regulating protein stability (Ciechanover and Schwartz, 1994). However, in our previous study, USP33 interacts with Robo1 and is required for Slit-induced Robo1 redistribution to the plasma membrane without affecting the total Robo1 protein level. Data in this study show that USP33 interacts and stabilizes Robo1 protein level in lung cancer cells by inhibiting degradation via the ubiquitin proteasome pathway. USP33 mediates Slit signaling in lung cancer cells, and this requires the enzymatic activity of USP33, because expression of USP33 mutant (C163A) abolishes the Slit activity in lung cancer cells. Interestingly, four missense mutations in the catalytic domain of USP33 have been identified in lung cancer samples in the datasets from the cBioPortal for Cancer Genomics. It will be interesting to test whether these mutations affect the catalytic activity of USP33 in future studies. Our previous work on breast cancer and the current data from lung cancer suggest that USP33 may mediate Slit signaling in different cancer cells through different mechanisms.

In summary, our results showed that USP33 expression was significantly decreased in lung cancer tissues. Higher USP33 expression is associated with better prognosis. USP33 regulates Slit signaling by stabilizing Robo1 and is required for Slit inhibition of lung cancer cell migration. These findings support that USP33 is a previously unknown player in lung cancer and suggest that USP33 may be a new prognostic biomarker for lung cancer.

## Materials and methods

### Ethical statement

De-identified lung tumor pathological samples were collected after pathological diagnosis in Tangdu Hospital, Fourth Military Medical University, China. Specimens were obtained with informed consent following approved protocols and following institutional and national guidelines.

### Lung cancer tissues and cell lines

For mRNA extraction, samples were frozen in liquid nitrogen immediately after surgical removal. For immunohistochemistry, lung cancer samples were fixed in 10% neutral-buffered formalin overnight and then paraffin embedded sectioned, and stained with haematoxylin and eosin according to the standard protocol.

H1299 cells and HEK239 cells stably expressing Slit or control were cultured in Dulbecco’s modified Eagles medium supplemented with 10% (*v*/*v*) fetal bovine serum, 50 mg/mL penicillin/streptomycin, incubated at 37°C in a humidified atmosphere of 5% CO_2_ as described previously (Wu et al, 1999).

### Antibodies, reagents, and plasmids

Antibodies used were as follows: anti-USP33 (ProteinTech Group, Inc), anti-Robo (ProteinTech Group, Inc), anti-beta actin (ProteinTech Group, Inc), anti-Flag (Sigma Aldrich, Inc) and anti-HA (Covance). Reagents used were as follow: Cycloheximide (CHX) (Sigma Aldrich, Inc), MG132 (Sigma Aldrich, Inc), Lipofectamine 2000 (Invitrogen, Inc), and enhanced chemiluminescence reagent kit (ECL; Millipore). Human type II USP33 tagged with GFP at the N-terminus (GFP-USP33), GFP-USP33C163A mutant constructs, Robo-HA, Flag-Ub plasmids have been previously described (Yuasa-Kawada et al., 2009b).

### Transfection

Transient transfection with plasmid DNAs was carried out by using Lipofectamine 2000 (Invitrogen) according to the manufacturer’s instructions. siRNAs against USP33 (#1: 5′-UCUCGACAGUGGCUUAAUUAA-3′; #2: 5′-GGAUUCAGUUGGUGAAAUUAC-3′) and negative control siRNA (5′-CGUACGCGGAAUACUUCGATT-3′) were synthesized by GenePharma Co., Ltd, Shanghai, China. After overnight incubation, the culture medium was replaced with fresh Dulbecco’s modified Eagle’s medium containing 10% fetal bovine serum.

### Quantitative RT-PCR

Total RNA was extracted from lung cancer tissues using Trizol (Invitrogen, Inc). The amount and purity of RNA were measured spectrophotometrically on Ultrospec 5300 pro (Amersham Biosciences). Genomic DNA was removed from RNA samples by digestion with DNaseI, according to the manufacturer’s instructions. First strand cDNA was synthesized from 5 μg of total RNA using the oligo (dT) primers and Moloney Murine Leukemia Virus Reverse Transcriptase (M-MLV RT) (Invitrogen, Inc) according to the manufacturer’s instructions. Real-Time PCR was used to quantify transcript levels and performed on the ABI StepOne real-time PCR using Absolute Blue SYBR green PCR mix (Thermo Fisher Scientific). The following primers were used: USP33 (5′-TGTGATGCTTAGGCAAGGAG-3′ and 5′-GGCCCTCCACCATAAATAGA-3′); Robo1 (5′-GCATCGCTGGAAGTAGCCATACT-3′ and 5′-CTAGAAATGGTGGGCTCAGGAT-3′); GAPDH (5′-GGAGCGAGATCCCTCCAAAAT-3′ and 5′-GGCTGTTGTCATACTTCTCATGG-3′). We used a 2-step amplification (40 cycles of 95°C, 15 s; 60°C, 30 s; 72°C, 30 s; followed by melting temperature determination stage) and fold enrichment was calculated by the 2^−ΔΔCT^ method.

### Immunoprecipitation and Western blotting analyses

Immunoprecipitation and Western blotting analyses were carried out as previously published (Yuasa-Kawada et al., 2009a). Briefly, 48 h (for plasmids), or 72 h (for siRNA) after transfection, H1299 cell lysates were prepared with lysis buffer (0.5% NP-40, 50 mmol/L Tris [pH 7.5], 150 mmol/L NaCl, 1 mmol/L EDTA, 50 mmol/L NaF, 1 mmol/L Na_3_VO_4_, 1 mmol/L DTT, 1 mmol/L PMSF, cocktail). Cleared cell lysates were used for immunoprecipitation, and immunoprecipitated proteins were detected by Western blotting using corresponding antibodies as indicated.

### Immunohistochemistry

Immunohistochemistry was performed on 5 micron-thick, formalin fixed, paraffin-embedded tumor sections, which were initially deparaffinized, rehydrated and heated in citric acid (pH 6.0) for 20 min in a microwave oven. After antigen retrieval, endogenous peroxidase activity was blocked by 3% hydrogen peroxide for 10 min, followed by 30 min blocking incubation in goat serum (Histostain™-Plus Kits; ZSGB-BIO). The slides were then incubated overnight at 4°C with primary antibody against USP33. Next, the slides were incubated with biotinylated antibody (Histostain™-Plus Kits; ZSGB-BIO) for 30 min at room temperature. Finally, slides were visualized by 3,3-diaminobenzidine (DAB) staining. Stained slides were digitalized with Scanscope XT (Aperio Technologies, Vista, CA). To quantitate the state of USP33 protein expression, the mean percentage of positive tumour cells was determined in at least five random fields at 200× magnification in each section. The intensity of the USP33-immunoreaction was scored as follows: 0, negative; 1+, weak; 2+, moderate; and 3+, intense. The percentage of positive tumour cells and the staining intensity then were multiplied to produce the USP33-immunohistochemical staining score.

### Recombinant Slit2 production and wound healing assay

The HEK293 cells that produce full-length Slit2 proteins tagged with c-myc have been described (Wu et al., 2001). The cells were cultured in DMEM with 5% FBS. Slit2 was partially purified from the supernatants as described previously (Wu et al., 2001). The supernatant from parental HEK cells was used as mock control.

For wound healing assay, H1299 cells were seeded on photo-etched grid coverslips coated with collagen in 35 mm culture. Once the cells reached confluence, a wound area was carefully created by scraping the cell monolayer with a sterile 10 μl pipette tip. The cells were rinsed with PBS to remove detached cells and then allowed to grow in the presence of control- or Slit- containing media. Ten images were taken under an inverted microscope at 0 and 12–16 h following the wound formation. Cell migration was quantified by measuring the forward migration distance of cells from the original position of the wound formation.

### Analyses of USP33 gene expression in human cancer samples

Genetic alterations and mRNA expression changes of the USP33 were presented analyzed in the data from cBioPortal for Cancer Genomics (Gao et al., 2013). Correlations between lung cancer and USP33 gene expression were determined through analysis of Stearman, Su, Bhattacharjee, Landi and Hou lung cancer datasets, respectively, which are available through Oncomine (http://www.oncomine.org/) (Rhodes et al., 2004). The correlations between lung cancer patients’ survival and USP33 gene expression were analyzed using TCGA data from Cancer browser database (https://genome-cancer.ucsc.edu/) (Zhu et al., 2009) and the online KM-Plotter database (Gyorffy et al., 2010; Gyorffy et al., 2013).

### Statistical analysis

All grouped data are presented as mean ± SEM. To determine statistical significance, results were compared by means of paired *t*-test (for two samples) or by analysis of variance (ANOVA) (for more than two samples). For survival analyses, Kaplan-Meier curves were generated using Prism software and log-rank analysis performed. All experiments presented were repeated at least three times. *P* < 0.05 at the 95% confidence level was considered significant.

## Electronic supplementary material

Below is the link to the electronic supplementary material.

Supplementary material 1 (PDF 3256 kb)

## Abbreviations

DMEM: Dulbecco’s Modified Eagle’s medium
Na_3_VO_4_: sodium orthovanadate
NaF: sodium fluoride
NSCLC: non-small-cell lung carcinoma
SCLC: small-cell lung carcinoma
