# Supplementary material for: USP33, a new player in lung cancer, mediates Slit-Robo signaling
Source: Protein Cell. 2014 Jul 2;5(9):704–13. doi: 10.1007/s13238-014-0070-z (PMC4145083; doi:10.1007/s13238-014-0070-z)
Supplement: Supplementary file 1 — Supplementary material 1 (PDF 3256 kb) [file 13238_2014_70_MOESM1_ESM.pdf]

SUPPLEMENTAL INFORMATION

Supplemental Information includes one table and four figures.

Table S1. Demographic data of lung cancer patients

Variables		Number
Sex	Male	21
	Female	4
Age (year)	<50	5
	50-60	12
	>60	8
Histology	SCLC	2
	Squamous cell carcinoma	12
	Adenocarcinoma	10
	Large cell carcinoma	1
Stage	I	3
	II	9
	III	10
	IV	3

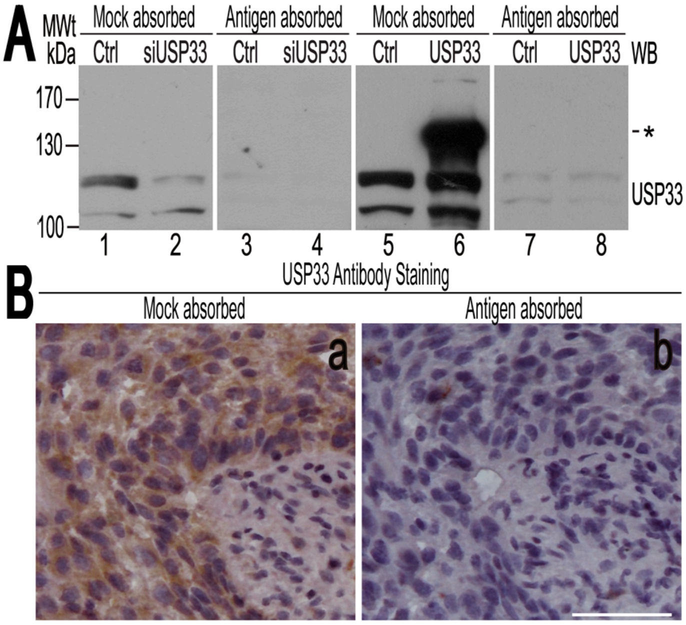

Figure S1. Specificity of polyclonal USP33 antibody.

(A) Western blot analysis of extracts from H1299 cells transfected with Ctrl siRNA or siUSP33 (lanes 1-4) or plasmids expressing the vector only (Ctrl) or USP33-GFP (lanes 5-8) using polyclonal USP33 antibody that was blocked with BSA (Mock absorbed) or the same antibody pre-incubated with the USP33 antigen (Antigen absorbed). The "*" marks the position of USP33-GFP. (B) Immunohistochemical staining of lung cancer tissue using the mock absorbed anti-USP33 antibody (a) or the antigen absorbed anti-USP33 (b). Bar: 50um.

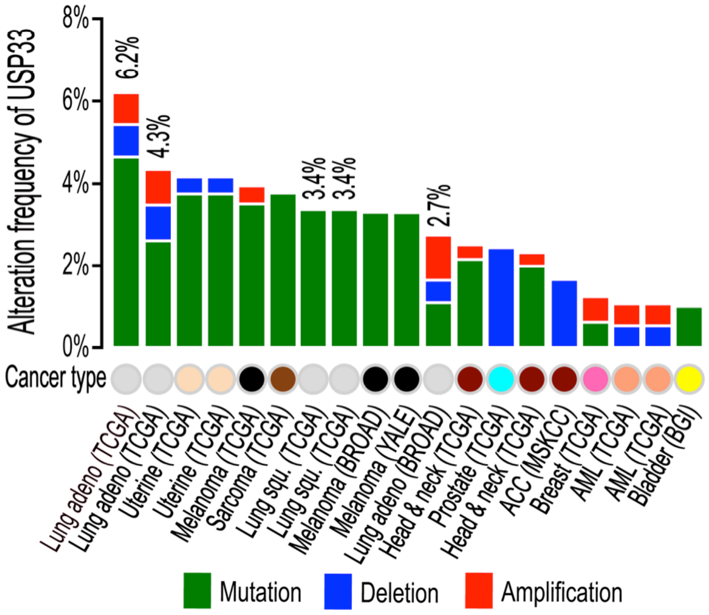

Figure S2. Frequency of genetic alterations of the human USP33 gene in different types of tumor samples (TCGA) as plotted using cBioPortal tools.

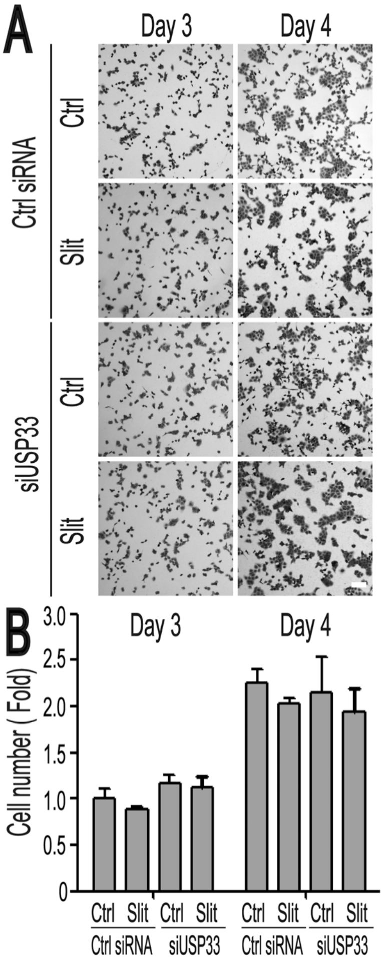

Figure S3. Cell proliferation was not affected by Slit treatment or USP33 downregulation in H1299 cells.

H1299 cells were transfected with Ctrl siRNA or siUSP33 and treated with control or Slit after transfection. The cells were fixed by in methanol and stained with 0.1% crystal violet solution. The number of cells in each well was counted. (A) Representative images of cells stained by crystal violet in each groups. (B) Quantification of cells numbers in each group. The cell numbers are not significantly different between each group at day 3 or day 4 after transfection. Scale bar: 200μm.

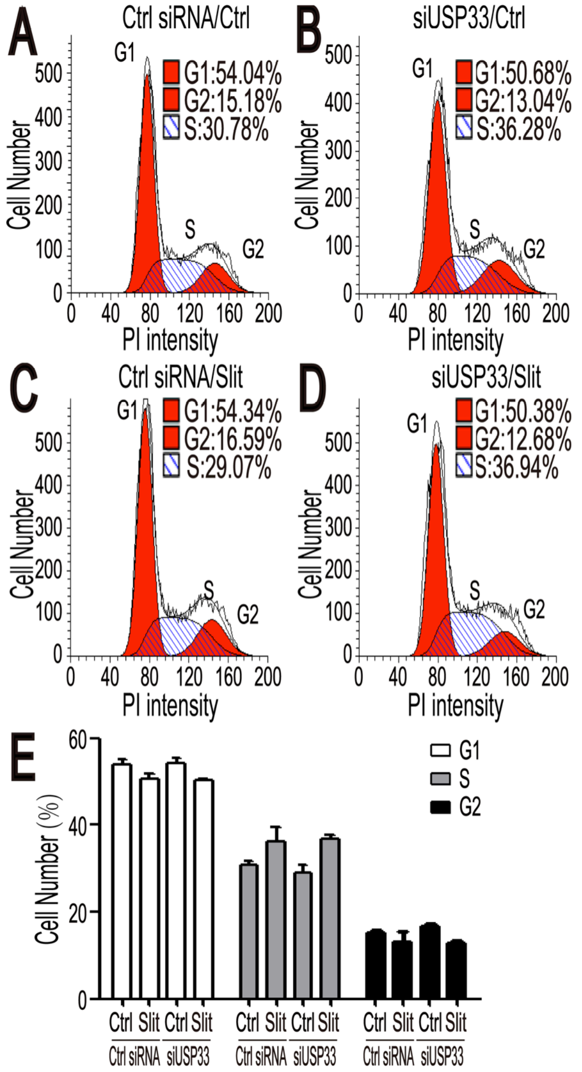

Figure S4. FACS analyses of cell cycle in H1299 cells transfected with Ctrl siRNA or siUSP33 and treated with the control or Slit.

H1299 cells were transfected with Ctrl siRNA or siUSP33 for 72 hrs and then treated with the control or Slit containing medium for 24 hours. The cells were fixed by the 70% ice-cold ethanol and stained with propidium iodide for DNA contents. Cells in different phases of cell cycle were detected by fluorescence-activated cell sorting (FACS). (A and B) H1299 cells transfected with Ctrl siRNA or siUSP33 in the control treated groups. (C and D) H1299 cells transfected with Ctrl siRNA or siUSP33 in the Slit treated groups. (E). The fractions of cells in each phase of cell cycle (G1, S and G2, as indicated as % cells) were determined using Flowjo cell cycle analysis software. The ratio of cells in each phase was represented on the Y-axis. There is no significant difference among different groups. The data represent three independent experiments.
